# Screening of postpartum depression among new mothers in Istanbul: a psychometric evaluation of the Turkish Edinburgh Postnatal Depression Scale

**DOI:** 10.1186/s13104-020-05196-x

**Published:** 2020-07-28

**Authors:** Perran Boran, Ahmed Waqas, Öykü Özbörü Aşkan, İrem Topçu, Tugay Dogan, Atif Rahman

**Affiliations:** 1 Division of Social Pediatrics, Department of Pediatrics, School of Medicine, Marmara University, İstanbul, Turkey; 2 Human Development Research Foundation, F-7/4, Islamabad, Pakistan; 3 Institute of Population Health, University of Liverpool, The Waterhouse Building, Dover St, Liverpool, L3 5DA UK

**Keywords:** Edinburgh Postnatal Depression Scale, Turkey, Validation, Reliability

## Abstract

**Objective:**

This study is the first concerted effort to ascertain factor structure of EPDS using evidence based analytical techniques. It is the most widely used scale for assessing postpartum depression in Turkey, and yet no investigations have been conducted to assess it factor structure. This study was conducted from April 2012 to April 2018 at the Marmara University Hospital operating under the name of Marmara University Pendik Training and Research Hospital in Istanbul Turkey.

**Results:**

A total of 1700 women were included in this study, who responded to the EPDS, in addition to demographic characteristics and well-being of their offspring. A total of 1615 mothers provided adequate data for inclusion in analysis. Standardized Chronbach’s alpha for EPDS was 0.81 with corrected item-total correlations ranging from 0.35 to 0.62. Parallel analysis, MAP Velicer Test and Hull’s method dictated retaining of one factor structure. All the items revealed adequate communalities (> 0.20) except item 2 (enjoyment) and item 10 (self-harm). Their communalities were 0.16 and 0.19, however, these items were not dropped. All of the items yielded moderate to strong factor loadings. Minimum factor loading was for item 2 (0.40) and highest for item 8 (0.71).

## Introduction

Postpartum depression (PPD) is a debilitating common mental disorder and constitutes a major global health concern [1]. Recent estimates place the prevalence of postpartum depression at 19.8% (19.5–20.0) post-birth [1]. These high prevalence estimates have also been reported among Turkish mothers, ranging from 21 to 36% [2–4]. Two meta-analysis including over 50 studies, estimate the weighted mean prevalence of PPD to be around 24% in Turkey [5, 6]. Studies from around the world have shown association of PPD with poor child outcomes. For instance, mothers with moderate to severe depressive symptoms at 9 months postpartum report children with shorter stature [average 0.26 cm shorter; 95% CI 5 cm, 48 cm] than their counterparts; and this effect persists for first 6 years of child’s life [7]. Moreover, postpartum depressive symptoms are also associated with poorer overall child cognitive and physical development [8].

One of the most frequently used instrument used for screening of PPD is the Edinburgh Postnatal Depression Scale (EPDS) [9]. It is a 10-item self-administered scale, developed for detection of PPD in community and primary care settings. Developed on the basis of Research Diagnostic Criteria for depressive illness obtained from Goldberg’s Standardized Psychiatric Interview [10], it has demonstrated adequate criterion, face and factor validity [10]. The scale is easily comprehensible, completed in a short time (~ 5 min) and has a simple scoring pattern. More than 90% of the studies on PPD, in Turkey, utilize the EPDS scale [5, 6]. A Turkish translation of the EPDS (Additional file 1) was found reliable among a Turkish population in the year 2004, albeit reporting poor criterion validity [4]. No investigations so far have been conducted to ascertain dimensionality, or factor structure of EPDS in Turkey. Therefore, this study aims to validate the EPDS using exploratory and confirmatory factor analytical techniques, to provide a robust evidence for its factor validity and reliability in a Turkish population.

## Main text

### Methods

#### Study design & setting

This psychometric validation study is part of a prospective birth cohort study of newborn infants followed up at the well-child outpatient clinic, Marmara University Pendik Training and Research Hospital from the year 2012 to 2018, located in Pendik, Turkey. Since 2012, the outpatient clinic provides care for newborns of the mothers who give birth at the university hospital’s maternity clinic. They receive a pamphlet with information regarding how to make an appointment at the well child outpatient clinic. Each term newborn (≥ 37 weeks of gestational age) is generally scheduled for a first appointment at 1 month of age and is then followed up regular basis, up to 5 years of age. Mothers are screened for postpartum depression at 1 month well child visit using the EPDS [4]. Ethical approval for this study was taken from the Ethical Review Committee at the Marmara University, Turkey. Written informed consent was taken from all participating mothers.

#### Statistical analysis

Recent evidence for minimum sample size recommendations, however, suggest that psychometricians should consider number of number of factors, variables to factor ratio (p/f), strength of communalities as well as level of criterion for a particular scale [11]. Therefore, keeping recent literature on EPDS, a minimum sample size of 50 seems to be appropriate for wide communalities. p/f (10), unidmensional factor structure and excellent criterion (0.98) [9, 10, 12, 13].

All analyses were conducted using SPSS (v.25) and FACTOR software [14]. Visualization of histogram, Q–Q plots as well as values of skewness and kurtosis were used to assess normality in EPDS scores of participants. Floor and ceiling effects were considered significant if ≥ 20% either scored the lowest or maximum score on EPDS [15]. Internal consistency of the EPDS was tested using the Cronbach’s alpha value, Mislevy & Block and McDonald’s Omega, which was considered adequate at ≥ 0.7 [16–18]. Convergent validity was assessed performing item-scale Pearson’s product moment correlations corrected for overlaps, considered adequate at ≥ 0.2 for all items [19]. To assess the factor structure and dimensionality of EPDS in present sample, exploratory factor analyses was conducted using three techniques namely: Principal Axis Factoring (PFA), Principal Component Analysis (PCA) and Maximum Likelihood technique (ML). We chose to run PFA in addition to ML, because PFA is more robust when the data violates assumptions of multivariate normality [20]. Total number of factors to retain was judged using several criteria including Cattell’s Scree plot, parallel analysis based on minimum rank factor analysis, MAP Velicer Test and Hull’s method [21]. Thereafter, suitability of each item to include in the final scale was assessed using several criteria: (a) communality (≥ 0.2) and (b) factor loadings ≥ 0.32.

Confirmatory factor analysis was further run to analyze the goodness of fit of the factor structure of EPDS. Several goodness of normed and non-normed fit indices were utilized including comparative fit index (CFI), normed fit index (NFI), Tucker-Lewis index (TLI), incremental fit index (IFI). While absolute fit indices included the goodness-of-fit index (GFI) and adjusted goodness-of fit index (AGFI) as well as root mean square error of approximation (RMSEA), root mean squared residual (RMR), standard root mean squared residual (SRMR) [22]. Cut-off values for goodness of fit indices were > 0.90, RMSEA at < 0.08 or not significantly greater than Kelley’s criterion, and < 0.10 for SRMR [22].

### Results

#### Demographic characteristics

EPDS data of 1614 mothers with a mean age of 28.87 years (5.46) was included in the exploratory and confirmatory factor analyses (missing data n = 86). Mean number of years of education received by mothers were reported to be 8.64 (3.83). Most of the mothers were housewives 1314 (81.4%), 22 (1.4%) were unqualified workers, 100 (6.2%) low to middle quality workers, 136 (8.54%) were qualified government workers and 42 (2.6%) were professional workers.

#### Face and content validity

The participants generally reported good comprehensibility of the EPDS scale at the time of administration, pointing to a good face validity. Content validity however, was not assessed as it was done in a previous publication that details the forward and backward translation process and criterion validity of the questionnaire [4].

#### Descriptive statistics

**Fig. 1:**
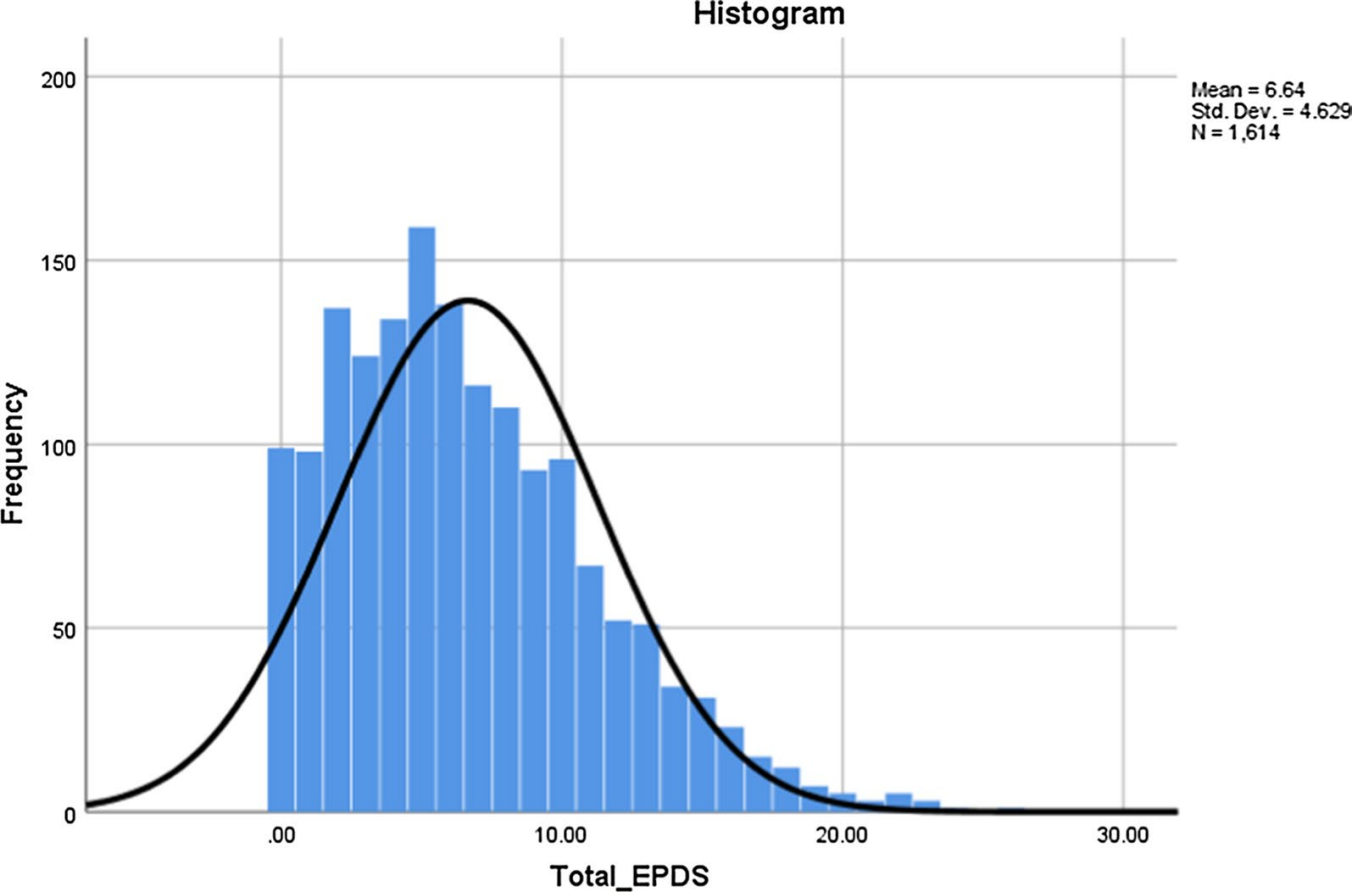
Histogram presenting distribution of EPDS scores among new Turkish mothers. The histogram shows that distribution of total scores on EPDS was mildly skewed among Turkish mothers

Mean score on Edinburgh Postnatal Depression Scale (EDPS) was 6.64 (4.63). Visualization of histogram and normal Q–Q plot revealed some degree of non-normality where distribution of total scores on EPDS was mildly skewed (0.78) and non-kurtotic (0.42) (Fig. 1). Mean scores on individual items ranged from 0.08 for item 10 exhibiting fewer symptoms of suicidality to 1.39 for item 3 exhibiting self-blaming or guilt among mothers. Furthermore, symptoms of anxiety (Item 4) and panic (Item 5) were most reported by the mothers (Table 1).

**Table 1:** Internal consistency and item-total correlations for individual items on EPDS

Item	Mean	SD	Scale mean if item deleted	Corrected item-total correlation	Cronbach’s alpha if item deleted
1. I have been able to laugh and see the funny side of things	0.16	0.433	6.48	0.409	0.785
2. I have looked forward with enjoyment to things	0.17	0.448	6.47	0.346	0.789
3. I have blamed myself unnecessarily when things went wrong	1.39	0.954	5.25	0.455	0.779
4. I have been anxious or worried for no good reason	1.16	0.934	5.49	0.545	0.765
5. I have felt scared or panicky for no very good reason	1.02	0.980	5.63	0.483	0.775
6. Things have been getting on top of me	0.83	1.003	5.82	0.507	0.772
7. I have been so unhappy that I have had difficulty sleeping	0.62	0.857	6.02	0.474	0.775
8. I have felt sad or miserable	0.71	0.802	5.93	0.619	0.756
9. I have been so unhappy that I have been crying	0.50	0.707	6.15	0.578	0.763
10. The thought of harming myself has occurred to me	0.08	0.339	6.57	0.369	0.790

Overall, a total of 310 (19.2%) of the mothers screened positive for depressive symptoms. Symptoms of guilt were reported by 1246 (77.20%), anxiety 1120 (69.39%), panic 979 (60.66%), sadness 893 (55.34%), poor coping 743 (46.03%), lack of sleep 660 (40.89%), crying spells 650 (40.27%), anhedonia 240 (14.87%), decreased mood 230 (14.25%), and suicidal ideation 90 (5.58%). Floors and ceiling effects were not evident in total scores of EPDS scale with less 20% of the respondents scoring either the lowest or highest of the possible scores on EPDS. A total of 99 (6.1%) respondents reported the lowest score on EPDS while only 1 (0.1%) reported the highest scores on it. This indicates that psychometric testing using EPDS was fit to measure depressive symptoms and responsive to change without being impaired by floor and ceiling effects.

#### Reliability and convergent validity

Standardized Chronbach’s alpha for EPDS was 0.81with corrected item-total correlations ranging from 0.35 to 0.62. All the items had adequate item-total correlations, revealing no multicollinearity or singularity and were retained at this stage for exploratory factor analyses. Moreover, inter-item correlation matrix was run to ascertain convergent validity with all items exhibiting a correlation value of 0.2 with at least one other item (Table 1). Moreover, other tests for reliability yielded adequate reliability of EPDS as assessed by McDonald’s Omega (0.81) and Mislevy & Bock [18] reliability estimate of 0.83.

#### Factor validity

Prior to running exploratory factor analyses, sampling adequacy was ascertained using the KMO statistics, yielding a good sampling adequacy (0.88), along with a significant Bartlett test of sphericity (χ2 = 3456.03, p < 0.001). Thereafter, observation of correlation matrix revealed that all EPDS items had yielded a correlation > 0.2, at least with one other item. Item 9 (crying spells) yielded highest correlation value of 0.54 with Item 8 (sadness). Thus, there were no issues of multicollinearity in the data. Sampling adequacy for each item was measured using KMO measure of sampling adequacy obtained in anti-image correlation matrix. It ranged from 0.863 (Item 1) to 0.915 (Item 7), therefore, yielding marvellous to meritorious KMO values for individual items. Therefore, all items were taken into exploratory factor analyses.

The criteria for determining the number of factors to retain was multifaceted and dependent on several factors including Eigen values > 1, Cattell’s Scree plot as well as more advanced methods such as parallel analysis, Hull’s method and MAP Velicer test. A total of 2 factors yielded an Eigen Value greater than 1.0 in present analysis. The first factor had an Eigen value of 3.68 explaining a variance of 36.77% while the second factor had an Eigen value of 1.10 leading to a cumulative 47.79% of variance explained by the two factors. However, Cattell’s Scree plot favoured a one-dimensional model, demonstrating a sharp drop in Eigen value, from first to second factor. This uni-dimensionality was further confirmed in more advanced statistical analyses such as Parallel analysis. Parallel Analysis was run based on minimum rank factor analysis with 500 replicates (Timmerman and Lorenzo-Seva 2011). This simulation revealed that the mean of random percentage of variance (18.1%) explained by second factor was greater than the percentage of variance obtained through EFA (12.9%). These were further confirmed by Minimum Average Partial Velicer test as well as the Hull Method.

All the items revealed adequate communalities (> 0.20) except item 2 (enjoyment) and item 10 (self-harm). Their communalities were 0.16 and 0.19, however, these items were not dropped. All the items yielded moderate to strong factor loadings (Table 2). Minimum factor loading was for item 2 (0.40) and highest for item 8 (0.71).

**Table 2:** Factor loadings for individual items obtained with PFA and ML

Statements	PAF	ML	PCA	Communalities
1. I have been able to laugh and see the funny side of things	0.483	0.477	0.55	0.234
2. I have looked forward with enjoyment to things	0.400	0.395	0.47	0.160
3. I have blamed myself unnecessarily when things went wrong	0.493	0.493	0.56	0.243
4. I have been anxious or worried for no good reason	0.592	0.591	0.65	0.350
5. I have felt scared or panicky for no very good reason	0.512	0.506	0.58	0.262
6. Things have been getting on top of me	0.567	0.562	0.63	0.322
7. I have been so unhappy that I have had difficulty sleeping	0.535	0.536	0.60	0.286
8. I have felt sad or miserable	0.714	0.721	0.74	0.510
9. I have been so unhappy that I have been crying	0.674	0.681	0.72	0.454
10. The thought of harming myself has occurred to me	0.434	0.435	0.50	0.188

#### Confirmatory factor analysis

Confirmatory Factor Analysis with the Exploratory Maximum Likelihood (ML) was run to confirm the goodness of fit for one-dimension structure of EPDS. It revealed that the one-dimension structure for EPDS yielded adequate values for all the indices representing the goodness of fit. It yielded a RMSEA value of 0.066 (< 0.08) which was not significantly greater than the cut-off value of 0.05. And according to Hair et al. (2010) and Hu and Bentler [22], revealed a good fitness of the model. All goodness of fit indices > 0.90 including CFI (0.93), TLI (0.91), GFI (0.99), AGFI (0.98), and GFI without diagonal values (0.97). Root Mean Square of Residuals (RMSR) was 0.047 which was not significantly larger than the expected mean value of RMSR for an acceptable model, as obtained by the Kelley’s criterion (4/√sample size).

#### Known group analysis with characteristics of mother

There was significant association of EPDS scores with improved housing index (r = 0.1, p < 0.05) and high income (r = 0.1, p < 0.05). There were no significant relationships between type of delivery (χ2 = 0.69, p > 0.05), mother’s education levels (p > 0.05) and age (p > 0.05).

## Discussion

The present study found EPDS to be a reliable and valid tool based on exploratory and confirmatory factor analyses in a large study sample with 19.2% of mothers screening positive for depression. Although EPDS is a globally used scale, previous analyses have mainly reported its criterion-validity and data on other aspects of validity especially the factory validity are lacking. This point has been emphasized in several meta-analyses in Africa, Europe and globally [9, 10, 13, 23, 24]. Akin to this, previous validation study of EPDS among Turkish population, only reported criterion validity [4]. However, measuring criterion validity as the sole measure of validation is inadequate, and it is very essential to conduct construct, content, convergent, and concurrent validity to ascertain the cross-cultural interchangeability for a particular psychometric instrument [25].

The importance of factor validation for EPDS has been emphasized in cross-cultural studies, reporting varying factor solutions for it. For instance, EPDS has exhibited a three factor structure comprising anhedonia, depression and anxiety among the Spanish population [26]. A varied model comprising of subscales of anhedonia, anxiety and low mood was shown as having best fit among Hungarian population [27]. In a similar vein, a three factor structure of EPDS comprising of anhedonia, anxiety and depressive mood has exhibit best model fit indices among the US and Japanese maternal population [28, 29]. Increasingly reports of heterogeneous factor structures are being reported in other countries [30, 31]. Therefore, it is important to test dimensionality of EPDS in different settings before implementing screening clinics for postpartum depression.

## Limitations

Inclusion of a large sample size from an entire district of Turkey favours adequate power of the study and generalizability of its results.

## Supplementary information

**Additional file 1:** Turkish Translation of Edinburgh Postnatal Depression Scale.

## Data Availability

All data associated with this manuscript are available on request to the corresponding author.

## Abbreviations

PPD: Postpartum depression
EPDS: Edinburgh Postnatal Depression Scale
PFA: Principle axis factoring
ML: Maximum likelihood
PCA: Principle component analysis
