# Supplementary material for: Screening of postpartum depression among new mothers in Istanbul: a psychometric evaluation of the Turkish Edinburgh Postnatal Depression Scale
Source: BMC Res Notes. 2020 Jul 28;13:355. doi: 10.1186/s13104-020-05196-x (PMC7390134; doi:10.1186/s13104-020-05196-x)
Supplement: Supplementary file 1 — Additional file 1: Turkish Translation of Edinburgh Postnatal Depression Scale. [file 13104_2020_5196_MOESM1_ESM.doc]

EDINBURGH DOĞUM SONRASI DEPRESYON ÖLÇEĞİ

Sizin son hafta içindeki duygularınızı öğrenmek istiyoruz. Böylelikle size daha iyi yardımcı olabileceğimize inanıyoruz. Lütfen, yalnızca bugün değil son 7 gün içinde, kendinizi nasıl hissettiğinizi en iyi tanımlayan ifadeyi işaretleyiniz.

### Son 7 gündür;

*1) Gülebiliyor ve olayların komik tarafını görebiliyorum.*

Her zaman olduğu kadar

Artık pek okadar değil

Artık kesinlikle okadar değil

Artık hiç değil

### Son 7 gündür;

*2) Geleceğe hevesle bakıyorum.*

Her zaman olduğu kadar

Her zamankinden biraz daha az

Her zamankinden kesinlikle daha az

Hemen hemen hiç

### Son 7 gündür;

*3) Birşeyler kötü gittiğinde gereksiz yere kendimi suçluyorum.*

Evet, çoğu zaman

Evet, bazen

Çok sık değil

Hayır, hiç bir zaman

### Son 7 gündür;

*4) Nedensiz yere kendimi sıkıntılı ya da endişeli hissediyorum.*

Hayır, hiç bir zaman

Çok seyrek

Evet, bazen

Evet, çoğu zaman

### Son 7 gündür;

*5) İyi bir nedeni olmadığı halde, korkuyor ya da panikliyorum.*

Evet, çoğu zaman

Evet, bazen

Hayır,çok sık değil

Hayır, hiç bir zaman

### Son 7 gündür;

*6) Her şey giderek sırtıma yükleniyor.*

Evet, çoğu zaman hiç başa çıkamıyorum

Evet, bazen eskisi gibi başa çıkamıyorum

Hayır,çoğu zaman oldukça iyi başa çıkamıyorum

Hayır, her zamanki gibi başa çıkabiliyorum

### Son 7 gündür;

*7) Öylesine mutsuzum ki uyumakta zorlanıyorum.*

Evet, çoğu zaman

Evet, bazen

Çok sık değil

Hayır, hiç bir zaman

Son 7 gündür

*8) Kendimi üzüntülü ya da çökkün hissediyorum.*

Evet, çoğu zaman

Evet, oldukça sık

Çok sık değil

Hayır, hiç bir zaman

Son 7 gündür

*9) Öylesine mutsuzum ki ağlıyorum.*

Evet, çoğu zaman

Evet, oldukça sık

Çok seyrek

Hayır, asla

Son 7 gündür

*10) Kendime zarar verme düşüncesinin aklıma geldiği oldu.*

Evet, oldukça sık

Bazen

Hemen hemen hiç

Asla
